# Supplementary material for: Boron isotopes in boninites document rapid changes in slab inputs during subduction initiation
Source: Nat Commun. 2022 Feb 22;13:993. doi: 10.1038/s41467-022-28637-6 (PMC8863828; doi:10.1038/s41467-022-28637-6)
Supplement: Supplementary file 1 — Supplementary Information [file 41467_2022_28637_MOESM1_ESM.pdf]

Supplementary Information

Boron isotopes in boninites document rapid changes in slab inputs during subduction initiation

Hong-Yan Li[1, 2, 3*], Xiang Li[1, 4*], Jeffrey G. Ryan[5*], Chao Zhang[6], Yi-Gang Xu[1, 2, 3]

[1] *State Key Laboratory of Isotope Geochemistry, Guangzhou Institute of Geochemistry, Chinese Academy of Sciences, Guangzhou 510640, China*

[2] *CAS Center for Excellence in Deep Earth Science, Guangzhou, 510640, China*

[3] *Southern Marine Science and Engineering Guangdong Laboratory (Guangzhou), Guangzhou, 511458, China*

[4] University of Chinese Academy of Sciences, Beijing 100049, China

[5] *School of Geosciences, University of South Florida, Tampa, FL 33620*

[6] *State Key Laboratory of Continental Dynamics, Department of Geology, Northwest University, Xi'an 710069, China*

* Corresponding authors:

Hong-Yan Li E-mail address: hongyanli@gig.ac.cn

Xiang Li E-mail address: lixiang@gig.ac.cn

Jeffrey G. Ryan E-mail address: ryan@usf.edu

1. Supplementary Methods

Samples examined in this study were selected from the working halves of cores for Holes U1439 and U1442, to represent both the natural chemical variability of the Exp. 352 boninites and to reflect overall core stratigraphy. Both fresh boninites that were selected specifically for these measurements, and larger 'POOL' low silica boninite (LSB) samples that were chosen for coordinated post-cruise measurement were analyzed. The major element and trace element abundances and Sr-Nd-Hf isotopic ratios of the 'POOL' samples may be found in Li et al.[1]. The methods described below include B isotopes for the 'POOL' samples and major elements, trace elements and Sr-Nd-Hf-B isotopes for the 'Non-POOL' samples.

1.1. Sample preparation

The fresh boninite samples were crushed into ~0.2 mm chips, examined and hand-picked under a binocular microscope for the freshest fragments. The handpicked rock chips were leached in 1% HCl and rinsed ultrasonically in deionized water, then dried and powdered to 200 mesh in an agate mill for major and trace elemental determination. The 'POOL' samples were cut on the ship, and powdered in agate and/or alumina onshore for coordinated measurements.

1.2. Major and trace element analyses

Trace element analyses on all the samples were performed at Guizhou Tongwei Analytical Technology Co., Ltd. on a Thermal X series 2 ICP-OES equipped with a Cetac ASX-510 AutoSampler[1]. Approximately 60 mg of each rock powder was dissolved in a Teflon bomb in a concentrated mixture of 4:1 doubly distilled

HF-HNO_3. Samples were heated in an oven at 185 ℃ for 3 days. The solutions were dried down, samples were re-dissolved in double distilled concentrated HNO_3, followed by 1:1 HNO_3, and dried again to convert fluorides to nitrates. Samples were ultimately dissolved in a 3ml 2M HNO_3 stock solution that was then diluted to 4000:1 in 2% HNO_3, and spiked with 12ppb ^{6}Li, 6ppb ^{61}Ni, Rh, In and Re, and 4.5ppb ^{235}U internal standards. The USGS reference material W-2a was used as reference standard and BIR-1, BHVO-2 and several other reference samples were crosschecked. Instrument drift and mass bias were corrected using these internal spikes and external monitors. The ICP-MS procedure for trace element analysis follows the protocols of Eggins et al.[2], with modifications as described in Kamber et al.[3] and Li et al.[4]. Based on results for rock standard BIR-1a, the analytical precision for the rare earth elements (REE) and most of the other species analyzed is ±1–5%.

Major element analyses were performed at the State Key Laboratory of Isotope Geochemistry, Guangzhou Institute of Geochemistry, Chinese Academy of Sciences (GIGCAS), using a Varian Vista Pro inductively coupled plasma atomic emission spectrometer (ICP-AES). The sample digestion procedure was similar to that for trace element determination, with the sample solutions ultimately diluted to 2000:1 in 3% HNO_3 before ICP-AES measurement. Details of the measurement techniques may be found in Li et al.[5]. The precision for major elements was better than 1%.

1.3. B-Sr-Nd-Hf isotope analyses

B-Sr-Nd-Hf isotope analyses were performed at the State Key Laboratory of

Isotope Geochemistry, GIG-CAS.

1.3.1. B concentration and isotope analyses

A subset of coarse grained fresh boninite samples and all of the 'POOL' samples were leached in 6 mL distilled 6 M HCl before sample digestion, following the methods of Li et al.[6], to remove seawater-derived boron (specifically B from marine carbonates and from secondary zeolites). For a subset of the samples, both leached and unleached materials were analyzed to assess the effects of seafloor alteration on B isotopic compositions.

Sample digestion and ion-exchange chromatography

~150 mg of each unleached rock powder was precisely weighed into a 15 mL pre-cleaned polypropylene (PP) tube for sample digestion. The leached residues of ~300 mg of rock powder were directly digested in 15 mL PP tubes, following the methods of Li et al.[6]. 200 μL 2% mannitol, 150 μL H_2O_2 and 1.5 mL 24 M HF were added to each tube. The tube was tightly capped, placed in a tube holder, and put on a hot plate at a temperature of 50-55 ℃ for 15 days for boron extraction. Solutions were then diluted with B-free Milli-Q deionized water to an HF molarity of 3 M for ion-exchange purification.

The samples were loaded onto pre-conditioned 20 ml columns with Bio-Rad AGMP-1 strong anion exchange resin for chromatographic purification. The column was eluted with 8 mL 3M HF followed by 8 mL B-free Milli-Q deionized water. Ti was extracted from the column using 48-66 mL 0.1 M HCl, and the extracts were measured in real time via ICP-AES to monitor the Ti levels until Ti in the elution was

< 2000 cps during ICP-AES measurement. Boron was extracted from the column in 12 mL 24 M HF, collected in a pre-cleaned 15 mL PP tube to which 200 μL 2% mannitol and 100 μL ethanediol were added to prevent volatilization losses of boron during drying.

<u>B concentration measurements</u>

A 2 mL aliquot of the above solution (unleached sample) collected from the column was diluted with 4 mL B-free Milli-Q deionized water for boron concentration measurement using the Varian Vista Pro ICP-AES, equipped with an HF-resistant Teflon spray chamber and an Al_2O_3 injector. Boron was measured using the 249.772 nm spectral line. B-5, JB-2, JB-3 and JR-2 were chemically prepared with the samples and used as external standards for calibrating B concentrations. The analytical precision for our boron concentration measurements was generally better than 5% (RSD).

<u>B isotope measurements</u>

The remaining sample solutions collected from the column were transferred into pre-cleaned 30 mL beakers, and then dried on a hot plate at temperatures < 55℃ for two to three days. The dried samples were re-dissolved in 1.0 mL B-free Milli-Q deionized water and transferred into 2 mL PP tube, and then were further diluted to a boron concentration of ~70-90 ng mL^{-1} for boron isotope measurement. The measurements were performed using a Finnegan Neptune MC-ICPMS in sample-standard-bracketing (SSB) mode. The SRM 951 standard dissolved in B-free Milli-Q deionized water was used as the bracketing standard, and the results of the

measured samples were expressed as $\delta^{11}B$ relative to SRM 951. Details of the analytical procedures of $\delta^{11}B$ are described by Li et al.[6]. The internal precision for $\delta^{11}B$ was better than ±0.05‰ (1SE), and external precision for $\delta^{11}B$ is better than ±0.40‰ (1SD) based on our long-term results for SRM 951. The standard reference samples B-5, B-6, JB-2, AGV-2, and JR-2 were repeatedly prepared and analyzed along our unknowns to monitor the quality of the B isotope measurements. Measured $\delta^{11}B$ values for the reference samples were: AGV-2: -4.36 ±0.68 ‰ (2SD, n=3); B-5: -4.71 ±0.49 ‰ (2SD, n=9); B-6: -2.86 ±0.62 ‰ (2SD, n=9); JB-2: +7.29 ±0.60 ‰ (2SD, n=9); JB-3: 6.74 ±0.09 ‰ (2SD, n=2); and JR-2: 3.10±0.77 ‰(2SD, n=11). The leached boninite sample U1442A-31R-1 was prepared and analyzed with two sets of samples, yielding +1.32 ±0.04 ‰ (SE) and +1.38 ±0.05 ‰ (SE), respectively. For most 'Non-POOL' samples, leached and unleached $\delta^{11}B$ were similar within error, though in a few cases changes of up to ±1‰ were observed (Supplementary Data 1). We use the leached $\delta^{11}B$ if available in the diagrams of the text.

1.3.2. Nd-Hf isotope analyses

Sample digestion

Approximately 100 mg of rock powder was weighed into pre-cleaned 15 mL teflon beakers with 2 mL concentrated HF and 1 mL concentrated HNO_3. The beaker was tightly capped on a hot plate at ~120 ℃ for 7 days. Then the sample solution was evaporated to incipient dryness and a further 1 mL of concentrated HNO_3 was added. The beaker was tightly capped again and put onto the hot plate overnight to

re-dissolve the sample. After that, the solution was evaporated again, re-dissolved with 1 mL of 6 M HCl, and capped on a hot plate at ~ 100 ℃ for ~ 4 h to ensure complete digestion.

Nd-Hf ion-exchange chromatography

The sample solution in 6 M HCl was evaporated and then re-dissolved with 1.5 mL of 2.5 M HCl. The sample solution was centrifuged and the supernatant was loaded onto pre-conditioned 2 mL AGW50-X12 cation exchange resin. The high field strength elements (HFSEs) were extracted from the column with 4mL of 2.5 M HCl. Then 8 mL of 2.5 M HCl were added to extract most of the major elements, such as K, Mg, Fe and Ca et al. Finally the rare earth elements (REEs) were extracted with 14 mL of 6 M HCl.

The eluted REEs were dried on a hot plate and re-dissolved with 0.5 mL 0.25 M HCl. To further separate the Nd from other REEs, Ln resin was used with di (2-ethylhexyl) orthophosphoric acid (HDEHP) from Eichrom Company, USA. After sample loading onto the pre-conditioned column, the inner column wall was washed three times with 1 mL of 0.25 M HCl. Then, the column was eluted with 3.5 mL of 0.25 M HCl. Finally, Nd was extracted from the column with 7 mL of 0.25 M HCl.

For Hf purification, Ln resin was used as same as Nd purification. The eluted solution containing HFSEs were directly loaded onto pre-conditioned Ln resin. Most matrix elements were eluted with 10 mL 4M HCl. Titanium was leached with 20 mL of 4M HCl + 0.1% H_2O_2. Then the column is treated with a further 20 mL of 1.2 M

HCl+0.06 M HF. Finally, Hf was extracted from the column with 3mL of 2 M HF.

Hf-Nd isotope measurements

The Nd and Hf isotopic ratios were measured on a Neptune MC-ICPMS in low resolution mode. Neodymium and Hf isotope ratios were monitored and corrected for mass bias using the values of $^{146}Nd/^{144}Nd$ = 0.7219 and $^{179}Hf/^{177}Hf$ = 0.7325, respectively. The Nd isotopic ratios are reported relative to $^{143}Nd/^{144}Nd$ of JNdi-1=0.512115. The rock standard BHVO-2 was repeatedly analyzed with chemical treatment in separated aliquot for each analysis, yielding a results of $^{143}Nd/^{144}Nd$=0.512986$\pm$4 (1SD, n=2), in agreement with recommended values of $^{143}Nd/^{144}Nd$ = 0.512990$\pm$10[7]. Analysis of rock standard JB-3 and W-2A gave $^{143}Nd/^{144}Nd$=0.513043$\pm$5 and 0.512529$\pm$5 (SE), respectively, in agreement with recommended values of 0.513048 and 0.512519 (GeoRem). The Hf isotopic ratios are reported relative to $^{176}Hf/^{177}Hf$ of JMC 14374=0.282189 (corresponding to JMC475 of 0.282158). Analysis of rock standard BHVO-2 and BCR-2 yield $^{176}Hf/^{177}Hf$ = 0.283097$\pm$4 and 0.282858$\pm$3 (SE), in agreement with recommended values of $^{176}Hf/^{177}Hf$ = 0.283105$\pm$11 and 0.282870$\pm$8[8], respectively.

1.3.3. Sr isotope analyses

Sample digestion and Sr ion-exchange chromatography

The sample powders except for glass samples were leached using distilled 6 M HCl to eliminate carbonate infillings and possible alteration products following the

methods described by Weis et al. [9] before digestion. The leached samples were then centrifuged, and the residues were rinsed with deionized water three times and evaporated to dryness for digestion. Each of the sample power containing with ~ 800 ng Sr was weighed into a 7 ml Teflon beaker for sample digestion. The weight of the leached sample was estimated with a recovery of 50% for Sr concentration after leaching. The detail digestion process was the same as that for Hf-Nd isotope analysis. Before column chemistry, the sample solution in 6 M HCl was evaporated to dryness then treated with 1mL concentrated HNO_3 to convert the cations into nitrate-form. After that, the sample solution was dried again and re-dissolved with 0.25 ml 3M NHO_3 for Sr purification. The sample solution was loaded onto a column filled with 0.25 ml Spec Sr resin that was pre-conditioned with 3M NHO_3. Matrix elements were eluted with 6.5 ml 3M HNO_3. Sr was finally extracted from the column with 3 mL 0.05M HNO_3.

<u>Sr isotope measurements</u>

Sr isotopes of the 'Non-POOL' samples were analyzed in the same batch as the 'POOL' samples. The Sr isotopic ratios were measured on a Thermo Triton TIMS, and $^{87}Sr/^{86}Sr$ was corrected for instrumental mass fractionation by normalizing to $^{88}Sr/^{86}Sr$ = 8.375209. The SRM 987 reference standard analyzed along with the samples gave $^{87}Sr/^{86}Sr$ = 0.710235±14 (SD, n = 7). The Sr isotope results are reported relative to SRM 987 of $^{87}Sr/^{86}S$ = 0.710248. The rock standard BHVO-2, AGV-2, JB-3 and W-2A were chemical treated and measured along with the samples to monitor the quality of Sr analysis. They gave $^{87}Sr/^{86}Sr$ of 0.703506±5, 0.703976

± 7, 0.703432 ± 7, and 0.706952 ± 7 (2SE), respectively, in agreement with recommended values of 0.703479 ± 20, 0.703981 ± 9[7], 0.703446, and 0.706965 (GeoRem), respectively.

The Sr-Nd-Hf isotopes of the samples were corrected to 50 Ma. εNd_i and εHf_i were calculated with a CHUR value of 0.512630 for $^{143}Nd/^{144}Nd$ and 0.282785 for $^{176}Hf/^{177}Hf$[10].

1.4. Pseudosection calculations

All modeling were computed in the Na_2O-CaO-K_2O-FeO-MgO-Al_2O_3-SiO_2-H_2O-TiO_2-O_2 (NCKFMASHTO) component system. XFe^{3+} (= $(Fe^{3+}/(Fe^{3+}+Fe^{2+}))$) was set as 0.12 to keep consistent with the oxidation state of MORB[11, 12]. Pseudosection calculations were performed using the Perple_X program[13, 14] (version 6.9.1) with the internally consistent thermodynamic dataset hp62ver.dat[15]. The following solid-solution models were used: olivine[16]; epidote[15]; silicate melt, augite, hornblende[17]; garnet, orthopyroxene, biotite, muscovite, chlorite[18]; *C*1 plagioclase, K-feldspar[19]; spinel[20]; ilmenite[21]. Other endmembers such as quartz, rutile and titanite were considered as pure phases. To evaluate the system source composition, H_2O content, mineral assemblages, and the melting extent, three representative samples were selected for thermodynamic modelling: high Ba/Nb amphibolite sample WT28A-5, low Ba/Nb amphibolite sample WT32 [22], and average group II gabbro components of the Atlantis Bank Gabbroic Massif (IODP Hole U1473A, SW Indian Ridge[23]). To reveal the influence

of source compositions on the ratio of trace elements (e.g., Ba/Nb and Sr/Nd), the calculation is designed to achieve 10% melting by changing M_{H2O} for all the three representative samples at 9 kbar and 900 ℃.

High Ba/Nb amphibolite sample WT28A-5: The temperature of fluid-absent solidus increases from 850 ℃ to 1040 ℃ in a narrow M_{H2O} range (3-2.3 mol. %). At 9 kbar, 900 ℃ and M_{H2O} = 4.81 mol%, the assemblage is made up of hornblende (47.1 wt%), augite (21.7 wt%), plagioclase (15.3 wt%), potassium feldspar (4.6 wt%), titanite (0.6 wt%), melt (10.7 wt%).

Low Ba/Nb amphibolite sample WT32: The M_{H2O} range (2.2-1.5 mol. %) of fluid-absent solidus is much lower than sample WT28A-5. At 9 kbar, 900 ℃ and M_{H2O} = 4.42 mol%, the assemblage is made up of hornblende (34.6 wt%), augite (36.9 wt%), plagioclase (16 wt%), titanite (2.3 wt%), melt (10.2 wt%).

Atlantis Bank Gabbro: The temperature of fluid-absent solidus varies from 850 ℃ to 1050 ℃ in a relative broader M_{H2O} range (3-0.7 mol. %). At 9 kbar, 900 ℃ and M_{H2O} = 4.96 mol%, the assemblage is made up of hornblende (38.3 wt%), augite (21.8 wt%), plagioclase (30 wt%), melt (10 wt%).

2. Supplementary Figures

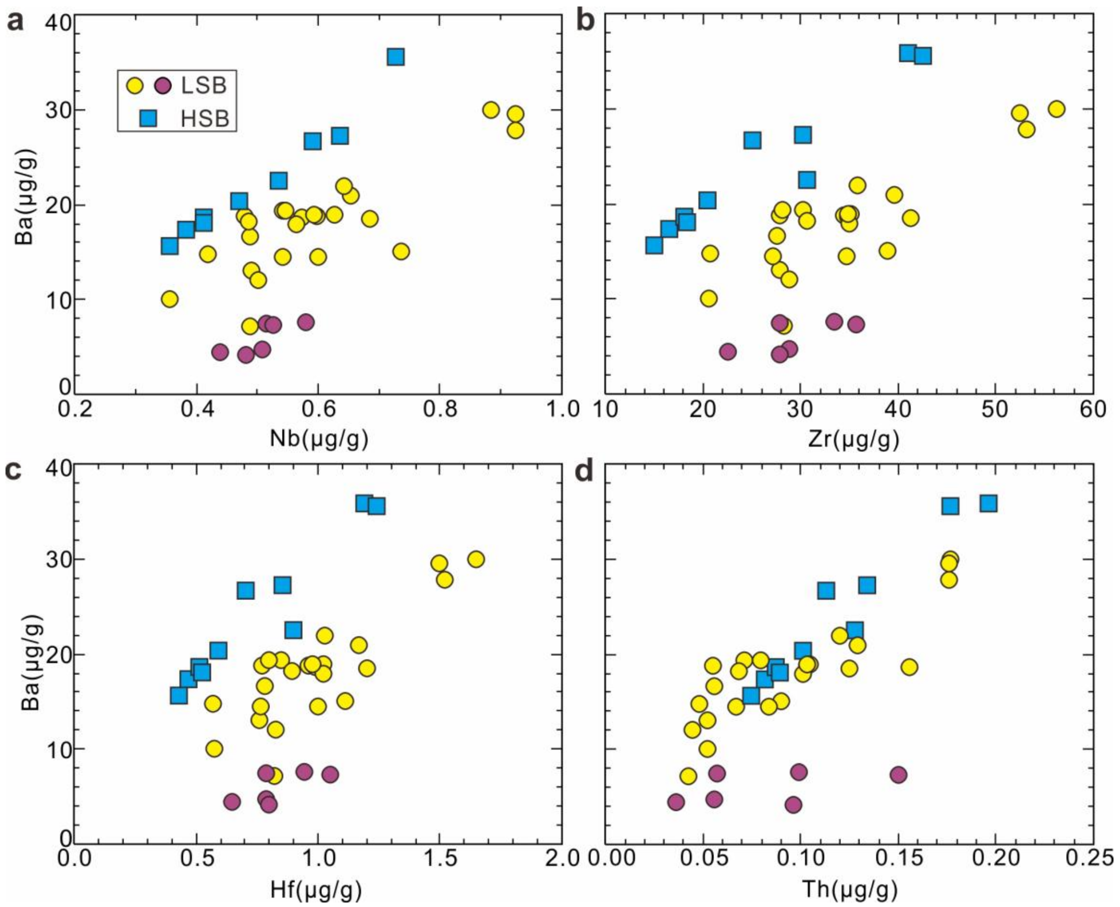

Supplementary Fig. 1 Elemental variations of the Expedition 352 boninites. Plots of Ba vs. **a** Nb, **b** Zr, **c** Hf and **d** Th diagrams for the Expedition 352 boninites. The positive correlations between Ba and the high field-strength elements indicate that they are primarily controlled by the magmatic process. LSB: low-silica boninite; HSB: high-silica boninite.

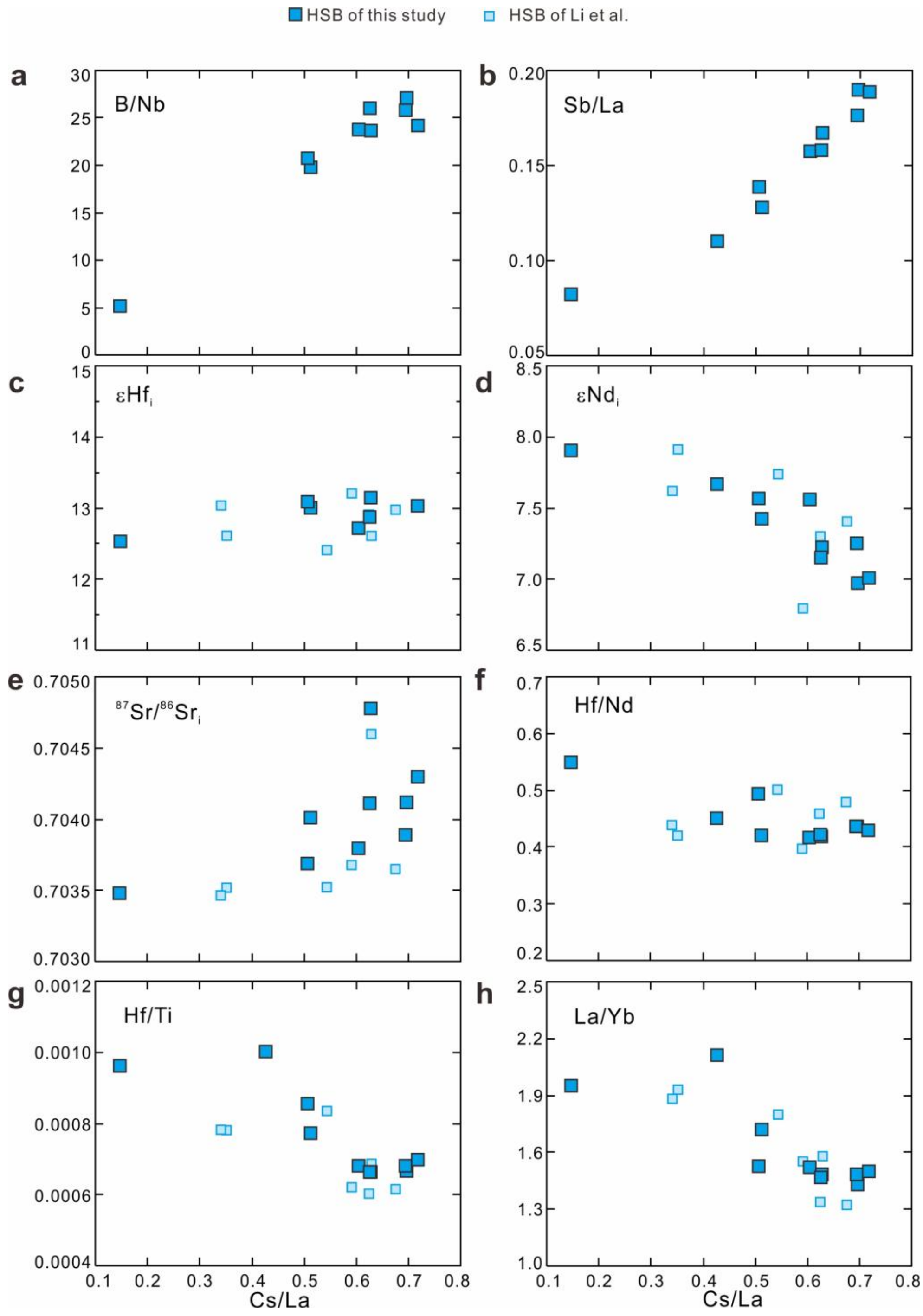

Supplementary Fig. 2 Chemical variations of the Expedition 352 high silica boninites. Plots of **a** B/Nb, **b** Sb/La, **c** εHf_i, **d** εNd_i, **e** $^{87}Sr/^{86}Sr_i$, **f** Hf/Nd, **g** Hf/Ti and **h** La/Yb vs. Cs/La diagrams for the Expedition 352 high silica boninites (HSB). HSB from Li et al.[1] are also plotted for comparison. Note that B, Sb, Cs, Sr and Nd are all synchronous enriched during HSB generation, whereas Hf shows no sign of further enrichment. Progressive HSB melt extraction from the mantle source may lower its Hf/Ti and La/Yb ratios.

3. Supplementary Tables

Supplementary Table 1 Geochemical compositions of different reservoirs

	$\delta^{11}B$ (‰)	B (μg/g)	Ba (μg/g)	Nb (μg/g)	Hf (μg/g)	Sr (μg/g)	Nd (μg/g)
**Average upper crust basalt**	+0.8±0.4[a]	26.2[a]	15.6[b]	2.89[b]	3.07[b]	109[b]	11.3[b]
**Average Izu-Bonin trench sediment**	-1.3±4[a]	94.2[c]	884[c]	5.22[c]	1.44[c]	136[c]	25.2[c]
**Basalt protolith low Ba/Nb amphibolite**		6.21[d]	29.76[d]	2.89[b]	3.07[b]	220.4[d]	11.3[b]
**Basalt protolith high Ba/Nb amphibolite**		17.31[e]	124.82[e]	2.89[b]	3.07[b]	407.9[e]	11.3[b]
**Average Gabbro**			2.4[f]	0.3[f]	0.8[f]	160.6[f]	3.4[f]

Data Source: a, Smith et al.[24]; b, Super composite composition of ODP Site 801[25]; c, Plank[26]; d, calculated according the B/Nb, Ba/Nb and Sr/Nd of the low Ba/Nb metamorphic sole amphibolite beneath the Oman ophiolite (samples WT32[22]); e, calculated according the B/Nb, Ba/Nb and Sr/Nd of the high Ba/Nb metamorphic sole amphibolite beneath the Oman ophiolite (samples WT28A-5[22]); f, average group II gabbro components of the Atlantis Bank Gabbroic Massif (IODP Hole U1473A, SW Indian Ridge[23]).

Supplementary Table 2 Partition coefficients used for hornblende-bearing granulite melting calculation

	Amphibole[a)]	Plagioclase[b)]	Clinopyroxene[c)]	Orthopyroxene[c)]	Titanite[d)]
B	0.011	0.175	0.17	0.0003	0.0003
Ba	0.152	0.2	0.00015	0.0036	0.003
Nb	0.492	0.005	0.005	0.0007	4.82
Hf	1.112	0.0005	0.42	0.017	1.58

Sr	0.339	0.833	0.111	0.0021	5.88
Nd	1.121	0.043	0.27	0.004	23.6

a) The value for B is an average from Tiepolo et al. [27]. The values for Ba, Nb, Hf, Sr and Nd are from Nandedkar et al. [28] at 0.7 GPa and 920℃.

b) The partition coefficient for B is average value from Bindeman and Davis [29]; Ba, Nb and Hf values are estimated based on Norman et al.[30] and Dunn and Sen [31]; Sr values are calculated according to the equation of Blundy and Wood [32] and corrected to pressure of 0.9 GPa and temperature of 900 ℃ based on the experimental results of Vander Auwera et al. [33], assuming plagioclase An of 0.85 given the experimental results of Koepke et al.[34]; Nd values are calculated according to the equation of Bédard [35], assuming pressure of 0.9 GPa and temperature of 900 ℃.

c) The partition coefficients for clinopyroxene are from Run R79 and those for orthopyroxene are from Run 1949 of Adam and Green [36].

d) The values for B and Ba are according the results of experiment rb21-1, and Nb, Hf, Sr and Nd values are according the results of experiment rb31-3 from Tiepolo et al.[37].

Supplementary References

1. Li, H. Y. et al. Radiogenic isotopes document the start of subduction in the Western Pacific. *Earth Planet. Sci. Lett.* **518**, 197–210 (2019).
2. Eggins, S. M. et al. A simple method for the precise determination of ⩾40 trace elements in geological samples by ICPMS using enriched isotope internal standardisation. *Chem. Geol.* **134**, 311-326 (1997).
3. Kamber, B. S. , Greig, A. , Schoenberg, R. & Collerson, K. D. A refined solution to earth's hidden niobium: implications for evolution of continental crust and mode of core formation. *Precambrian Res.* **126**, 289-308 (2003).
4. Li, B. P. et al. ICP-MS trace element analysis of Song dynasty porcelains from Ding, Jiexiu and Guantai kilns, north China. *J. Archaeol. Sci.* **32**, 251-259 (2005).
5. Li, X. H. et al. Geochemical and Sr-Nd isotopic characteristics of late Paleogene ultrapotassic magmatism in southeastern Tibet. *Int. Geol. Rev.* **44**, 559-574 (2002).
6. Li, X. et al. High-precision measurement of B isotopes on low-boron oceanic volcanic rock samples via MC-ICPMS: Evaluating acid leaching effects on boron isotope compositions, and B isotopic variability in depleted oceanic basalts. *Chem. Geol.* **505**, 76-85 (2019).
7. Weis, D. et al. High-precision isotopic characterization of USGS reference materials by TIMS and MC-ICP-MS. *Geochem. Geophys. Geosyst.* **7**, Q08006 **(**2006).
8. Weis, D. et al. 2007. Hf isotope compositions of US Geological Survey reference materials. *Geochem. Geophys. Geosyst.* **8**, (2007).
9. Weis, D., Kieffer, B., Maerschalk, C., Pretorius, W. & Barling, J. High-precision Pb-Sr-Nd-Hf isotopic characterization of USGS BHVO-1 and BHVO-2 reference materials. *Geochem. Geophys. Geosyst.* **6**, Q02002 (2005).
10. Bouvier, A., Vervoort, J. D., & Patchett, P. J. The Lu-Hf and Sm-Nd isotopic composition of CHUR: Constraints from unequilibrated chondrites and implications for the bulk composition of terrestrial planets. *Earth Planet Sci. Lett.* **273**, 48–57 (2008).
11. Cottrell, E. & Kelley, K. A. The oxidation state of Fe in MORB glasses and the oxygen fugacity of the upper mantle. *Earth Planet. Sci. Lett.* **305**, 270–282 (2011)
12. Palin, R. M. et al. High-grade metamorphism and partial melting of basic and intermediate rocks. *J. Metamorph. Geol*. **34**, 871–892 (2016).
13. Connolly, J. A. D. Computation of phase equilibria by linear programming: A tool for geodynamic modeling and its application to subduction zone decarbonation. *Earth Planet. Sci. Lett.* **236**, 524–541 (2005).
14. Connolly, J. A. D. Multivariable phase diagrams: an algorithm based on generalized thermodynamics. *Am. J. Sci*. **290**, 666–718 (1990).
15. Holland, T. & Powell, R. An improved and extended internally consistent thermodynamic dataset for phases of petrological interest, involving a new equation of state for solids. *J. Metamorph. Geol.* **29**, 333–383 (2011).
16. Holland, T. J. B. & Powell, R. An internally consistent thermodynamic dataset for phases of petrological interest. *J. Metamorph. Geol*. **16**, 309–343 (1998).

17. Green, E. C. R. et al. Activity–composition relations for the calculation of partial melting equilibria for metabasic rocks. *J. Metamorph. Geol.* **34**, 845–869 (2016).
18. White, R. W., Powell, R., Holland, T. J. B., Johnson, T. E. & Green, E. C. R. New mineral activity-composition relations for thermodynamic calculations in metapelitic systems. *J. Metamorph. Geol.* **32**, 261–286 (2014).
19. Holland, T. J. B. & Powell, R. Activity–composition relations for phases in petrological calculations: an asymmetric multicomponent formulation. *Contrib. Mineral. Petrol.* **145**, 492–501 (2003).
20. White, R. W., Powell, R. & Clarke, G. L. The interpretation of reaction textures in Fe-rich metapelitic granulites of the Musgrave Block, Central Australia: constraints from mineral equilibria calculations in the system. *J. Metamorph. Geol.* **20**, 41–55 (2002).
21. White, R. W., Powell, R., Holland, T. J. B. & Worley, B. A. The effect of TiO_2 and Fe_2O_3 on metapelitic assemblages at greenschist and amphibolite facies conditions: mineral equilibria calculations in the system K_2O–FeO–MgO–Al_2O_3–SiO_2–H_2O–TiO_2–Fe_2O_3. *J. Metamorph. Geol.* **18**, 497–511 (2000).
22. Ishikawa, T., Fujisawa, S., Nagaishi, K. & Masuda, T. Trace element characteristics of the fluid liberated from amphibolite-facies slab: Inference from the metamorphic sole beneath the Oman ophiolite and implication for boninite genesis. *Earth Planet. Sci. Lett.* **240**, 355–377 (2005)
23. Zhang, W. Q., Liu, C. Z. & Dick, H. J. Evidence for Multi-stage Melt Transport in the Lower Ocean Crust: the Atlantis Bank Gabbroic Massif (IODP Hole U1473A, SW Indian Ridge). *J. Petrol.* **61**, egaa082 (2020).
24. Smith, H. J., Spivack, A. J., Staudigel, H. & Hart, S. R. The boron isotopic composition of altered oceanic crust. *Chem. Geol.* **126**, 119–135 (1995).
25. Kelley, K. A., Plank, T., Ludden, J. & Staudigel, H. Composition of altered oceanic crust at ODP Sites 801 and 1149. *Geochem. Geophys. Geosyst.* **4**, 8910 (2003).
26. Plank, T. The Chemical Composition of Subducting Sediments. In Treatise on Geochemistry (2nd, ed. Rudnick, R. L.) **4**, 607–629 (Elsevier, Oxford, 2014).
27. Tiepolo, M., Oberti, R., Zanetti, A., Vannucci, R. & Foley, S. F. Trace-element partitioning between amphibole and silicate melt. Rev. *Mineral. Geochem.* **67**, 417–452 (2007).
28. Nandedkar, R. H., Hürlimann, N., Ulmer, P. & Müntener, O. Amphibole–melt trace element partitioning of fractionating calc-alkaline magmas in the lower crust: an experimental study. *Contrib. Mineral. Petrol.* **171**, 1–25 (2016).
29. Bindeman, I. N., & Davis, A. M. Trace element partitioning between plagioclase and melt: investigation of dopant influence on partition behavior. *Geochim. Cosmochim. Acta* **64**, 2863–2878 (2000).
30. Norman, M., Garcia, M. O. & Pietruszka, A. J. Trace-element distribution coeffi cients for pyroxenes, plagioclase, and olivine in evolved tholeiites from the 1955 eruption of Kilauea Volcano, Hawai'i, and petrogenesis of differentiated rift-zone lavas. *Am. Mineral.* **90**, 888–899 (2005).
31. Dunn, T. & Sen, C. Mineral/matrix partition coeffi cients for orthopyroxene,

plagioclase, and olivine in basaltic to andesitic systems: A combined analytical and experimental study. *Geochim. Cosmochim. Acta* **58**, 717–733 (1994).

32. Blundy, J. D. & Wood, B. J. Crystal-chemical controls on the partitioning of Sr and Ba between plagioclase feldspar, silicate melts, and hydrothermal solutions. *Geochim. Cosmochim. Acta* **55**, 193–209 (1991).
33. Vander Auwera, J., Longhi, J. & Duchesne, J. C. The effect of pressure on D_{Sr} (plag/melt) and D_{Cr} (opx/melt): implications for anorthosite petrogenesis. *Earth Planet. Sci. Lett.* **178**, 303–314 (2000).
34. Koepke, J., Feig, S. T., Snow, J. & Freise, M. Petrogenesis of oceanic plagiogranites by partial melting of gabbros: an experimental study. *Contrib. Mineral. Petrol.* **146**, 414–432 (2004).
35. Bédard, J. H. Trace element partitioning in plagioclase feldspar. *Geochim. Cosmochim. Acta* **70**, 3717–3742 (2006).
36. Adam, J. & Green, T. Trace element partitioning between mica-and amphibole-bearing garnet lherzolite and hydrous basanitic melt: 1. Experimental results and the investigation of controls on partitioning behaviour. *Contrib. Mineral. Petrol.* **152**, 1–17 (2006).
37. Tiepolo, M., Oberti, R. & Vannucci, R. Trace-element incorporation in titanite: constraints from experimentally determined solid/liquid partition coefficients. *Chem. Geol.* **191**, 105–119 (2002).
