# Supplementary material for: Boron isotopes in boninites document rapid changes in slab inputs during subduction initiation
Source: Nat Commun. 2022 Feb 22;13:993. doi: 10.1038/s41467-022-28637-6 (PMC8863828; doi:10.1038/s41467-022-28637-6)
Supplement: Supplementary file 3 — Description of Additional Supplementary Files [file 41467_2022_28637_MOESM3_ESM.pdf]

Description of Additional Supplementary Files

File name: Supplementary Data 1.

Description: Major elements, trace elements and Sr-Nd-Hf-B isotopes for the Expedition 352 boninites.
